# Functionally Designed Nanovaccines against SARS-CoV-2 and Its Variants

**DOI:** 10.3390/vaccines12070764

**Published:** 2024-07-12

**Authors:** Yue Xi, Rongrong Ma, Shuo Li, Gang Liu, Chao Liu

**Affiliations:** 1 State Key Laboratory of Stress Biology and Fujian Provincial Key Laboratory of Innovative Drug Target Research, School of Pharmaceutical Sciences, Xiamen University, Xiamen 361102, China; 32320221154501@stu.xmu.edu.cn (Y.X.); 32620221150854@stu.xmu.edu.cn (R.M.); 21620211153243@stu.xmu.edu.cn (S.L.); 2 State Key Laboratory of Molecular Vaccinology and Molecular Diagnostics, Center for Molecular Imaging and Translational Medicine, School of Public Health, Xiamen University, Xiamen 361102, China; gangliu.cmitm@xmu.edu.cn; 3 State Key Laboratory of Cellular Stress Biology, Innovation Center for Cell Biology, School of Life Sciences, Xiamen University, Xiamen 361102, China; 4 China Shenzhen Research Institute of Xiamen University, Shenzhen 518000, China

**Keywords:** nanovaccines, SARS-CoV-2, functionalization

## Abstract

COVID-19, generated by SARS-CoV-2, has significantly affected healthcare systems worldwide. The epidemic has highlighted the urgent need for vaccine development. Besides the conventional vaccination models, which include live-attenuated, recombinant protein, and inactivated vaccines, nanovaccines present a distinct opportunity to progress vaccine research and offer convenient alternatives. This review highlights the many widely used nanoparticle vaccine vectors, outlines their benefits and drawbacks, and examines recent developments in nanoparticle vaccines to prevent SARS-CoV-2. It also offers a thorough overview of the many advantages of nanoparticle vaccines, including an enhanced host immune response, multivalent antigen delivery, and efficient drug delivery. The main objective is to provide a reference for the development of innovative antiviral vaccines.

## 1. Introduction

**Figure 1:**
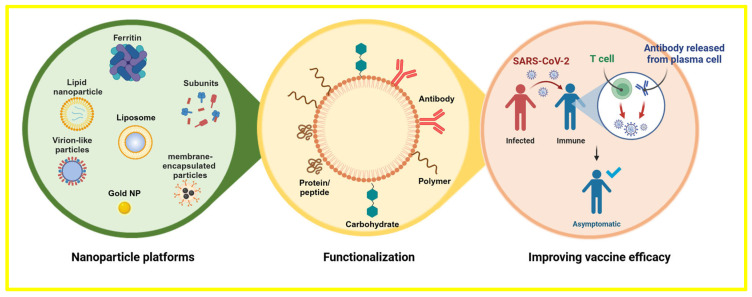
An outline of the subjects covered in this review, including standard nanoparticle platforms for antigen presentation that have been functionalized to improve the vaccination efficacy. Created with https://BioRender.com (accessed on 4 July 2024).

Global health has declined rapidly since the emergence of severe acute respiratory syndrome coronavirus 2 (SARS-CoV-2), which led to the COVID-19 pandemic. The viral outbreak has exhibited rapid expansion since its initial emergence in late 2019, leading to a substantial increase in morbidity and mortality rates on a global scale [1]. A primary concern regarding the outbreak is the emergence of multiple novel SARS-CoV-2 strains. During the virus’s reproduction process, spontaneous nucleotide changes occur in its genome, resulting in viral mutations, also referred to as variations. RNA viruses are more likely than DNA viruses to undergo mutations. It is normal for viruses to mutate during subsequent replication. It is estimated that the SARS-CoV-2 virus evolves at a pace of roughly 1.1 × 10^−3^ substitutions per site annually. This amount translates to a replacement occurring roughly every 11 days. Natural selection will cause a rise in the frequency of genetic modifications in the population, which are advantageous for viral replication, transmission, and immune evasion [2,3]. Creating new vaccination paradigms is necessary due to SARS-CoV-2’s high transmissibility, mutation rate, and immune evasion capabilities [4]. In addition to more conventional vaccination approaches like DNA- and vector-based vaccines and deactivated, live-attenuated, and regenerative protein vaccines, nanoparticle vaccines offer a singular opportunity to further immunization science and offer effective treatments for current and emerging pandemics [5,6]. Nanoparticles are adjustable, nanoscale particulate constructs that imitate the structural properties of natural viruses [7]. Because of their adaptive nature, they offer a viable foundation for the creation of vaccines for the next generation and can account for the variety and evolution of viral infections by eliciting a potent neutralizing antibody (nAb) response or broader antibody-based protection [8]. As per the latest data, around 190 prospective SARS-CoV-2 vaccines are presently under preclinical development, while 123 candidate vaccines are undergoing human clinical trials. More than 26 vaccine candidates based on nanoparticles are undergoing clinical trials, while another 60 are in the preclinical phase [9]. Nanotechnology has emerged as a promising avenue by which to combat viral infections, with nanovaccines using nanoparticles as carriers or adjuvants that show potential to enhance immunogenicity and facilitate targeted antigen delivery [10]. With extensive control over the vaccines’ features and reactions, these nanoparticles’ molecular structures can be precisely engineered to improve how well they interact with the immune system. Vaccines that are more stable and easier to administer can be created by taking advantage of the unique qualities of nanoparticles, such as their dimensions, forms, and surface chemistry. Vaccine durability and usability are important aspects to consider in light of the current global health crisis [11,12]. The development of quantitatively functionalized nanovaccines requires the careful consideration of several factors, including the choice of materials (e.g., lipid nanoparticles, protein-based nanoparticles, polymeric nanoparticles), the methods of antigen integration (e.g., adsorption, encapsulation, conjugation), and the routes of administration (e.g., intramuscular, intranasal, oral). These factors have the potential to significantly impact the ultimate vaccine formulation’s safety, as well as its effectiveness [13,14]. Furthermore, functional groups can be introduced to a nanoparticle’s surface to allow for prolonged release and tailored distribution, making vaccinations more effective [15]. In this study, we examine the recent developments in SARS-CoV-2 nanoparticle vaccinations from a clinical perspective, emphasize the advantages of functionalized nanovaccines over classical vaccine models, and examine the underlying principles of their design. Through this discourse, our objective is to underscore the possibilities of nanotechnology in creating the upcoming vaccine generation, which will tackle the intricacies of emerging infectious diseases like COVID-19 (Figure 1).

## 2. Nanotechnology for Vaccines

Nanovaccines (NVs) represent a breakthrough in immunization, offering new solutions to overcome the inherent limitations of traditional vaccine technologies, such as their often complex storage requirements, limited stability, and variable efficacy against rapidly evolving pathogens [16]. By exploiting the unique qualities of nanoparticles, nanovaccines can revolutionize infectious disease prevention and treatment, marking a significant advancement in public health and vaccine research [17]. There are already a number of nanoparticle-based vaccines that have been approved or successfully tested in clinical trials (Table 1). The capacity of nanoparticles to increase the immunogenicity of antigens is one of their greatest advantages. Nanovaccines are designed to resemble viruses in shape, form, and surface features, eliciting a more robust and natural immune response. This biomimetic technology significantly increases the efficacy of vaccinations by guaranteeing that antigens are successfully absorbed by the immune system and stimulating the activation of humorous and cellular immune system reactions [18,19]. Nanoparticle vaccines, designed to mimic the size (1–100 nm) and roughly spherical morphology of viral particles, are recognized favorably by the immune system, eliciting robust humoral immune responses. They play a crucial role in developing safe and reliable vaccine formulations that can swiftly advance to clinical trials [20]. Nanoparticles enhance the effectiveness and specificity of the immune response by accurately targeting adjuvants and antigens to specific cells or organs. Over time, the ability to control the release of these components further ensures sustained immune activation, thereby reducing the need for multiple doses. In addition, nanoparticles can serve as powerful adjuvants to boost the effectiveness of vaccination formulations without requiring an additional adjuvant component. The immune system perceives nanoparticles as alien, intensifying the body’s sensitivity to the vaccine antigen and initiating an innate immunological response [21,22,23]. Nanoparticles offer unrivaled versatility in vaccine design and formulation, offering diverse antigens, including proteins, peptides, nucleic acids, and polysaccharides. This adaptability makes them appropriate for a broad spectrum of disorders.

By carefully regulating the physicochemical characteristics of nanoparticles, such as their dimensions, electrical properties, and surface capabilities, vaccine delivery, immunogenicity, and stability can all be maximized. A crucial concern is vaccine durability during transportation and storage, particularly in settings with limited resources. Nanoparticles, especially those based on lipid nanoparticles or polymer systems, exhibit enhanced stability at various temperatures, thus reducing the reliance on cold chain logistics. The increased stability not only facilitates the global distribution of vaccines but also extends their shelf life, making vaccination campaigns more efficient and cost-effective. Non-specific vaccinations also reduce the possibility of non-specific immune activation and possible side effects through focused delivery and controlled release [24,25,26]. Using biocompatible and biodegradable materials to construct nanoparticles further reduces the risk of toxicity, making nanoparticle vaccines a safer alternative to some traditional vaccine formulations [27,28]. This rapid development contrasts that of traditional vaccines, which can take 15–20 years from concept to approval [29].

Nanovaccines can be delivered orally, intramuscularly, or transdermally. Their surfaces can be altered to boost immunity and encourage antigen-presenting cells (APCs) to absorb peptides unique to certain cell types [30,31,32,33]. Nanocarriers closely resemble the original virus in structure but are safe because they lack any viral genome, which prevents self-replication by the inoculant. Nanocarriers boost the immune system’s activity by shielding non-target areas from antigens until they reach their intended destinations [34]. In conclusion, nanocarriers offer numerous advantages over conventional vaccine technologies, including enhanced immunogenicity, targeted delivery, intrinsic adjuvant properties, multifunctionality, greater stability, and safety. With continued research and development in this area, nanovaccines are expected to provide more effective, efficient, and convenient solutions for the prevention and control of infectious and other diseases. A new age in vaccine creation will surely be ushered in by the ongoing investigation and improvement of nanoparticle-based vaccinations. This will significantly impact how vaccination programs and global health policies are shaped.

## 3. Functionalized Nanovaccines for SARS-CoV-2

**Figure 2:**
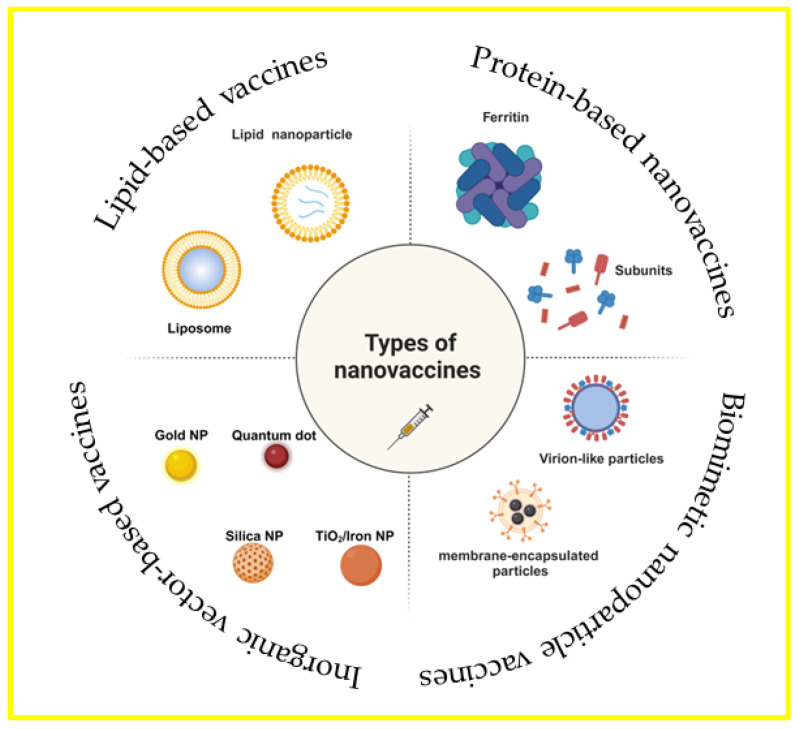
Vaccinations using biomimetic nanoparticles, vaccines based on proteins, vaccines based on lipids, and vaccines using inorganic carriers are the four general categories into which vaccine delivery methods utilizing nanoparticles can be divided, depending on the type of carrier used. There are distinct benefits specific to each carrier. Created with https://BioRender.com (accessed on 4 July 2024).

Worldwide, the development of vaccines against SARS-CoV-2, the organism that causes the novel coronavirus disease, is imperative. The use of nanovaccines, which improve vaccine administration and immunogenicity by incorporating nanotechnology, is a key tactic in the fight against this pandemic. Based on the carrier structure classification, vaccine delivery systems utilizing nanoparticles may be broadly divided into four categories: vaccines based on lipids, vaccines based on inorganic vectors, vaccines based on proteins, and vaccines employing biomimetic nanoparticles (Figure 2) [16,17,18]. The current phase of SARS-CoV-2 nanoparticle vaccine research is characterized by rapid innovation and growth, partly due to the urgent need for scalable and effective immunization strategies. Nanoparticle delivery platforms for drugs and vaccines have demonstrated promise in producing robust immune responses and offering stability and convenience of manufacturing—a vital combination for global immunization programs. Because of their adaptability, nanoparticle platforms may be quickly rearranged to include the sequences of recently identified strains, offering a way to update vaccinations in response to shifts in viral evolution [35,36]. Creating a nanoparticle vaccine to combat SARS-CoV-2 requires technological innovation to enhance its functional design, broaden its accessibility, and boost its adaptability to the virus’s ongoing evolution.

### 3.1. Lipid-Based Vaccines

Solid lipid nanoparticles (SLNs) and nanostructured lipid carriers (NLCs), two types of lipid nanoparticles with complex lipid ultrastructures, are distinct from regular liposomes in that their outer layer is more resilient and their center is less aqueous. Lipid nanoparticles (LNPs) are tiny, spherical lipid spheres that range in diameter from 50 to 1000 nm and have a compact hydrophobic core. Surfactant-stabilized lipids, emulsifiers, or both can be found in the cores of LNPs, together with desirable biomolecules such as drugs, peptides, proteins, or nucleic acids [37,38,39,40]. To tackle SARS-CoV-2, a novel class of nanovaccines called mRNA vaccines has been developed and implemented quickly. LNPs are employed in these vaccines to transport mRNAs encoding viral spike (S) proteins [41,42]. These mRNAs prompt host cells to produce virus-related proteins, which, in turn, elicit an immunological response [21,22]. The BNT162b2 vaccine from Pfizer Biotech and the mRNA-1273 vaccine from Moderna are two well-known examples of this approach. Both have significantly succeeded in Phase III clinical trials against COVID-19 [43,44].

**Figure 3:**
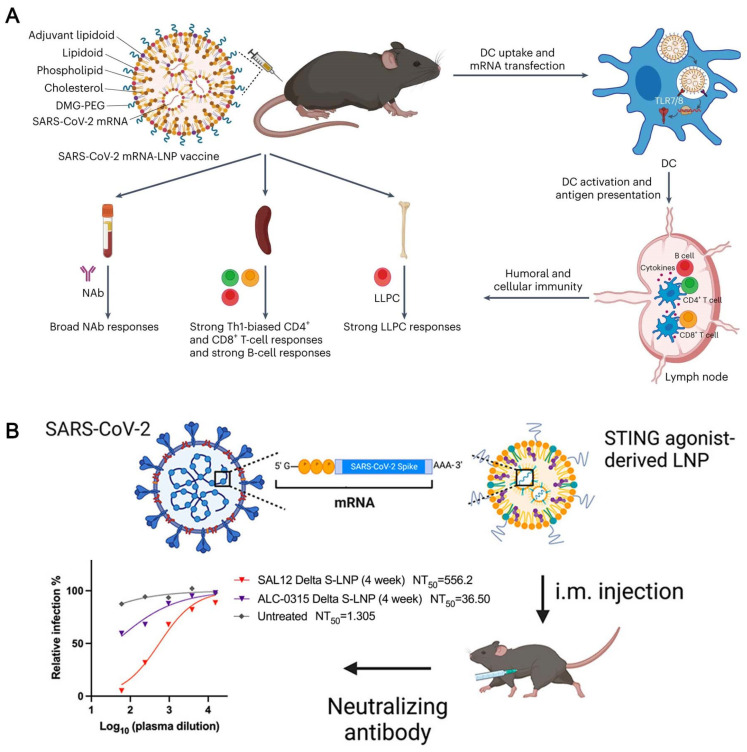
The lipid components in LNP nanovaccines are functionalized. (**A**) Adjuvant lipid replacement methods are effective in clinically significant mRNA-LNP vaccines [51]. (**B**) Innovative ionizable lipid compounds can activate the STING pathway and enhance the mRNA vaccine effect [55].

Recently, nanoimmunity by design has been presented as a way to precisely manage how nanomaterials affect an organism’s immune system. It is predicated on the logical design of functionalized nanomaterials with particular physicochemical properties. In this regard, applying nanostructures is a viable method of stimulating or suppressing immune responses [45]. Inducing T-cell responses and producing nAb against multiple SARS-CoV-2 variants has shown that mRNA vaccines are more effective than other types of vaccination. This could help to treat or prevent viral infections like those caused by the virus [46]. mRNA was first identified in 1961, but because of its high innate immunogenicity, ineffective in vivo transport, and sensitivity to enzymatic degradation, it was not widely used until recently in the production of vaccines [47]. The effectiveness of the SARS-CoV-2 mRNA vaccines has led to the recognition of lipid-nanoparticle-formulated mRNA vaccines as a potentially effective method of avoiding viral infections. Still, these alterations greatly attenuate the innate immune reactions necessary to coordinate strong adaptive immunity [48,49,50]. Notably, LNPs composed of ionizable lipids (or lipid-like) or PEGylated lipids, phospholipids, and cholesterol have become technologically advanced mRNA delivery systems. To further enhance the effectiveness of vaccines, researchers have become increasingly interested in the functional modification of LNP components, particularly lipid components. Michael J. Mitchell and colleagues have functionally modified the mRNA-LNP vaccine by developing a helper lipid, an LNP component, that can increase the adjuvant properties of the mRNA-LNP vaccine. Their findings demonstrate that supplementing a portion of the ionizable lipidoid with an adjuvant lipidoid enhances the mRNA transport and provides LNPs with toll-like-receptor-7/8-agonistic action, thereby considerably boosting the natural immunity of the SARS-CoV-2 mRNA-LNP vaccine, with animals displaying acceptable tolerance (Figure 3A) [51]. Crucially, the adjuvant lipidoid replacement approach is effective in clinically relevant mRNA-LNP vaccines, indicating its practical applicability. The antigen generated by the mRNA encoding the SARS-CoV-2 spike glycoprotein must be effectively transported and delivered to antigen-presenting cells to mount a successful immune response to SARS-CoV-2 infection. The activation of the innate immune system may aid in antigen presentation [52,53,54]. Yizhou Dong et al. have produced and packaged a collection of non-nucleotide STING agonist-derived amino lipids (SALs) to LNPs, allowing mRNA transport. The strong adjuvant effects of the SAL12-LNPs increased cytokine and antibody production. The SAL12-LNPs, loaded with mRNAs encoding the SARS-CoV-2 spike protein, generated potent and enduring IgG and neutralizing antibodies. Novel ionizable lipid compounds that trigger the STING pathway are expected to have a major impact on the development of mRNA vaccines (Figure 3B) [55].

### 3.2. Inorganic Vector-Based Vaccines

Inorganic nanoparticles, traditionally utilized in bio-imaging and cancer photothermal therapy, are now gaining interest for their application in vacuum canister delivery methods. Since most inorganic NPs exhibit immunomodulatory properties connected to the pyrroline-containing structural domain 3 (NLRP3)-mediated activation of NLR family inflammatory vesicles, they are very suitable for the creation of vaccinations. Once cellular uptake has occurred, these NPs boost the development of intracellular ROS. Afterward, lysosomal cathepsin B is released, triggering the creation of an inflammasome complex, which activates interleukin signaling and stimulates immune cells. Because of these characteristics, inorganic NPs are an excellent immunostimulant and antigen carrier for the identification, management, and avoidance of several illnesses, such as SARS-CoV-2 [56,57,58].

**Figure 4:**
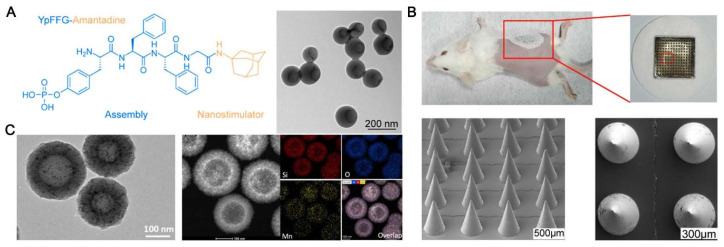
The inorganic carrier vaccine’s functional design to prevent SARS-CoV-2. (**A**) The cross-neutralization of SARS-CoV-2 strains stimulated by dimer RBD antigen is improved by amantadine-assembled nanostimulants [62]. (**B**) To incorporate the RBD of the SARS-CoV-2 spike protein into a microneedle patch vaccination, CpG 1018 and a graphene-based double adjuvant system are used [63]. (**C**) By interacting with Mn^2+^, the manganese–silicon nanoplatform (MnOx@HMSN) improves the adjuvant action of the CDN for SARS-CoV-2 vaccination and cancer [66].

To efficiently improve the function of monomeric materials, such as proteins, peptides, and tiny chemical compounds, an assembly method has been widely used in biomedicine. Numerous studies have also employed this technique to successfully deliver SARS-CoV-2-related antigens in conjunction with inorganic nanoparticles [59,60,61]. Ye Liu and colleagues have studied how an amantadine-assembled nanostimulator enhances the cross-neutralization against SARS-CoV-2 strains initiated by the dimeric receptor-binding domain (RBD) antigen. The establishment of a response related to T helper 1 is crucial for COVID-19 infection control (Figure 4A) [62]. Daxiang Cui et al. developed a graphene-oxide-based complex adjuvant nanovaccine (GCR) by rationally developing a CpG 1018 and graphene-oxide-based bi-adjuvant system to deliver the SARS-CoV-2 spike protein’s RBD. They have also created a microneedle patch vaccine (MGCR), which is based on GCR immunization. The outcomes showed that the RBD-specific GCR vaccination induced a notable CD8+ cell reaction in addition to a robust Type 1 immune response (Figure 4B) [63]. Due to their strong adjuvant properties and biocompatibility, silica nanoparticles have been extensively studied. They can be engineered to release their contents in a controlled manner, thereby strengthening the body’s defense against the antigen [64,65]. Manganese–silicon nanoplatform (MnOx@HMSN) was created by James J. Moon and colleagues that, by working in concert with Mn^2+^, improved the adjuvant effect of cyclic dinucleotides (CDNs) for SARS-CoV-2 vaccination and cancer. Mesopores of a large size are present in MnOx@HMSN. It is a versatile nanoplatform for vaccine application since it efficiently co-loads CDNs and peptide/protein antigens, enhances STING pathway activation, and enhances the dendritic cell production of type I interferons and other pro-inflammatory cytokines (Figure 4C) [66]. However, the lack of a comprehensive assessment of the long-term toxicity and biodegradation of inorganic nanoparticles following release into the environment is one potential drawback. While certain inorganic nanoparticles (NPs) are fully biodegradable, others may pose an unknown health risk and persist in the body for up to two years after renal clearance. Producing biocompatible nanoparticles that can eventually be eliminated is crucial for this reason.

### 3.3. Protein-Based Nanovaccines

Subunit vaccines can be designed to increase both the amount and quality of protection against the antigens that they contain through protein-based nanoparticle vaccination platforms. The biodegradability and adjustable characteristics of protein-based nanoparticles—polymeric NPs that are cheaply manufactured and frequently biocompatible—make them an especially alluring option. Many of these platforms can also serve as adjuvants by, among other actions, facilitating the multi-display of antigens and increasing the antigen exposure to immune cell constituents. Various distinct protein polymers, each with a different structure–function connection and advantages and disadvantages, are ideal for the production of vaccines. Numerous protein-based NPs have been assessed in preclinical and clinical settings since the COVID-19 pandemic’s emergence, as prompt vaccine rollout was necessary. Self-assembling protein nanoparticles (SAPNs) are nanostructures created when monomeric proteins oligomerize. Particles between 20 and 100 nm can be created by arranging SPANs as protein-building pieces. The production process of these proteins is frequently subject to strict monitoring and control to ensure that the final product is free from potentially contaminating impurities or pathogens and is therefore safe for use in biomedical applications. Recombinant technology allows for the precise production of specific proteins, which are often designed based on known gene sequences [67]. Greater control over immunogenicity can be achieved by using SPANs as antigen carriers that carry immunogenic material similarly targeted to cell receptors as viruses [68,69]. One such model is Novavax’s NVX-CoV2373, designed to facilitate a safe immune response using saponin-based Grid M adjuvants and spike proteins encapsulated in nanoparticles. Clinical preliminaries have shown the antibody’s important viability and impressive well-being profile [70]. Immunizations in light of viral vectors move the hereditary material of SARS-CoV-2 to cells utilizing safe infections, triggering the production of viral proteins and a resistant reaction. The use of viral vectors in vaccine distribution represents a type of nanotechnology, albeit not a true nanovaccine [71,72].

One of the most often employed naturally occurring proteins for this purpose is ferritin, which is generated in many species and regulates the availability of ferrous ions. It also has the potential to prevent the body from becoming reactogenic. Ferritins from several species have comparable architectural features, despite having varied amino acid sequences [73]. Additionally, since the recombinant ferritins generated in prokaryotic cells do not require additional translational modification, their production can be regulated. Ferritin is stable; it is resistant to urea, sodium hydroxide, and guanidinium chloride; and it can tolerate temperatures of up to 75 °C for ten minutes, in contrast to other proteins. Ferritin nanoparticles have an unusual architecture because they have two interfaces, one outside and one inside, which may each load a different molecule [74,75]. The external surface can be chemically or genetically functionalized, and high-affinity materials and other small molecules can be introduced into the internal cavity. Ferritin nanoparticles have recently been researched as a versatile tool for the delivery of antigens to immunize against viral infections. Spike-functionalized nanoparticles are used to combat SARS-CoV-2. Ferritin is expected to trigger an immune response that reacts with certain B cells by spreading different antigens over the particle [76].

**Figure 5:**
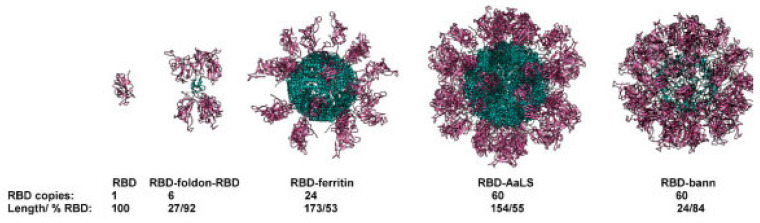
Combining a sequence of self-assembled scaffolds with protein antigens. The titer of RBD-specific antibodies, which also recognize spiking proteins, neutralizes the interaction between the spiking proteins and ACE2 receptors and offers protection in alternative infection assays (RBD domains are shown in violet and scaffold core in blue). This is significantly increased when RBDs are fused to the scaffolding structural domains [82].

Furthermore, research is currently being conducted on various protein-based nanoparticles, such as albumin, casein, and zein, for potential use in vaccination strategies (Figure 5). For instance, albumin and an adjuvant could be coupled to form a lipid tail to elicit an immunological response. In research, when this strategy was used with a CpG oligonucleotide in animals receiving subcutaneous immunization, their lymph nodes were exposed to a substantial 12-fold higher level than those receiving unmodified CpG26 [77]. This innovative technique is now being investigated in clinical trials to develop a cancer vaccine designed to target the lymph nodes. The use of protein nanoparticle vaccines against viruses including HIV, SARS-CoV-2, and influenza is also being investigated in ongoing clinical trials [78,79,80]; these trials have shown that the vaccinations are safe and can effectively produce neutralizing antibody responses in human subjects [81].

### 3.4. Biomimetic Nanoparticle Vaccines

Biomimetic nanoparticles are the next generation of drug therapies that mimic the physicochemical properties of pathogens while maintaining the surface properties of nanoparticles. This form of vaccination is safer and more effective than traditional vaccination. Drug delivery through biomimetic nanoparticles prevents the breakdown of antigens or adjuvants. Moreover, it avoids systemic toxicity to the body, such as free adjuvants that may produce adverse reactions including fever, drowsiness, diarrhea, and nausea. Virus-like particles (VLPs), membrane-camouflaging nanoparticles, and other biomimetic nanoparticles are currently available for vaccine production [18,83,84].

Virus-like particles, or VLPs, are synthetic viruses that lack viral nucleic acids and have nanoscale dimensions. VLPs function as adjuvants and vaccine carriers, such as virosomes. While delivering medications, proteins, peptides, and nucleic acids, VLPs are as effective as other vaccine carriers; however, to trigger a significant immune response, multiple doses and additional adjuvants are required [85,86,87,88]. Additionally, tailored administration is made possible by these VLP vaccines to ensure lymph node trafficking stability and a suitable size [31,32,33]. The hepatitis B virus (HBV) vaccine, which was initially approved in 1981, was the origin of VLP vaccinations. Its primary characteristic is that, despite lacking the genetic material of the original virus, it preserves and uses the capacity to form viral structural proteins to exhibit antigenic epitopes of the original virus, rendering it more generally accepted and safer. Commercial vaccines are available in a variety of VLP-based combinations to protect against a wide range of infectious diseases, such as HPV, malaria, and others [89,90,91,92]. The highly immunogenic VLP-based COVID-19 vaccines, which are abundant in antigenic epitopes of S, M, E, or N, directly crosslink B cell receptors to activate B cells, thereby inducing mice to develop neutralizing antibodies [93,94,95]. In preclinical experiments, the use of alum and Matrix-M1 adjuvants, along with vector lambda polymers containing full-length SARS-CoV and MERS-CoV S proteins, significantly increased the levels of coronavirus-neutralizing antibodies [96,97]. In addition to commercially available vaccines, another SARS-CoV-2 LYB001 RBD-based VLP vaccine (CHO cells) is in Phase I/II clinical trials in 2024 [98]. Gene fusion or the chemical conjugation of target antigenic epitopes with structural proteins from multiple sources to build chimeric vector lip products (VLPs) has become the standard approach, as not all viral antigenic proteins can be successfully constructed in vitro [99]. However, chimeric VLPs’ assembly is a highly unpredictable process. Issues like low yields, the inappropriate exposure of the injected antigens, and uneven and unstable assembly are frequently observed during development. A more reliable and adaptable chimeric VLP architecture is the modular molecular assembly method, in which the target antigen and VLP core are synthesized independently, and the antigen is covalently or non-covalently bonded to the surface of the pre-assembled core [100]. Antibody-dependent cytotoxicity may destroy cells with insert-ed or secreted antigenic proteins associated with their plasma membranes. Therefore, in situ, targeting specific immune cells is another direction for the development of next-generation vaccines, such as a DC-targeted virus-like particle (DC-VLPs) [19,101]. DC-VLPs are specialized nanoscale structures designed to specifically target DCs, key players in the immune system’s response to pathogens. DC-VLPs can be used as vaccine platforms by incorporating antigens that stimulate immune responses against specific pathogens. They efficiently deliver antigens to DCs, enhancing antigen presentation and immune activation. As DC-VLPs are non-infectious and lack viral genetic material, they offer a safe alternative to traditional viral vectors in vaccine development. Their structure and surface properties can be engineered to optimize their targeting, antigen presentation, and immune response. Advances in nanotechnology and protein engineering continue to improve the design and efficacy of DC-VLPs for targeted immunotherapies. In conclusion, DC-targeted virus-like particles represent a promising strategy in biomedical research for the enhancement of immune responses through targeted delivery to dendritic cells. Their development has the potential to improve vaccines and immunotherapies against a wide range of diseases [102,103].

**Figure 6:**
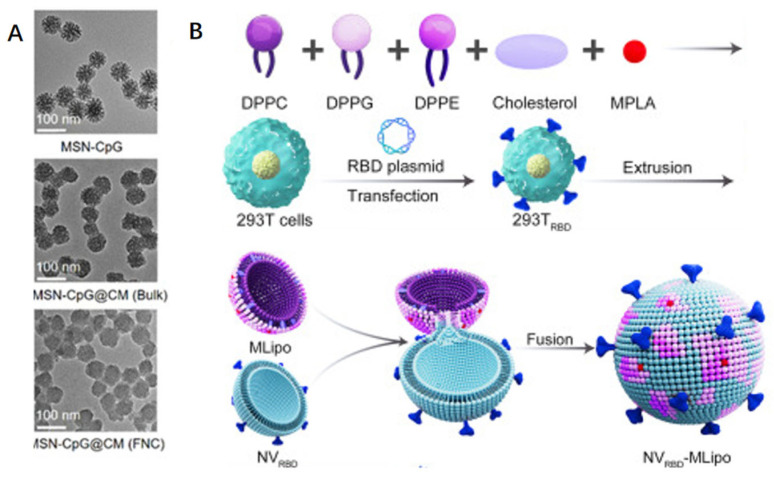
To achieve SARS-CoV-2-related antigen administration, biofilm camouflage nanoparticles can be used, classified into membrane fusion and encapsulation modes. (**A**) Biodegradable mesoporous silicon nanoparticles (MSNs) enclosed in transgenic cell membranes expressing SARS-CoV-2 RBD are coated with cytosine-phosphate-guanine oligodeoxynucleotide (CpG) [101]. (**B**) Nanovaccines produced by effectively fusing PS-biomimetic liposomes carrying MPLA (called MLipo) with transgenic NVs expressing RBD (named NVRBD) [19].

In a single delivery system, biomimetic nanoparticles integrate the unique capabilities of several nanomaterials with the biomimetic characteristics of diverse biofilms, including those derived from viruses, bacteria, hemoglobin, leukocytes, mesenchymal stem cells, dendritic cells, phagocytes, and platelets. Biofilm-encapsulated nanoparticles have become a proven and practical method to improve the distribution of various formulations within the last ten years [104,105]. In addition to helping to avoid some of the disadvantages of nanoparticles synthesized using traditional methods, such as poor colloidal stability, non-specific connective tissue accumulation, immune disapproval, and decreased systemic circulation, “cloaking” the synthesized nanoparticles with a natural membrane supports this straightforward top-down method [106]. Biomimicry makes the utilization of naturally occurring cell components like membranes and multivalent cell membrane indicators possible at the same time [107,108]. This includes targeting and immunomodulating cell surface markers [109,110,111,112]. According to a plethora of data, NPs encapsulated in erythrocyte cell membranes appear to have a longer half-life than PEGylated NPs, lasting 37 h instead of 16 h. These membrane proteins can disperse a nanocarrier as a “self” to boost its survival in the systemic circulation and evade reactions from the immune system [111,112]. Vaccine designs consisting of cell-membrane-encapsulated nanoparticles mostly consist of antigen-presenting cell membranes and adjuvant-carrying nanoparticles [113,114]. For example, a nanovaccine strategy was developed utilizing genetically engineered cell membranes expressing the SARS-CoV-2 RBD encapsulated with biodegradable mesoporous silica nanoparticles (MSNs), which encapsulated cytosine-phosphoryl-guanine oligodeoxyribonucleotides (CpGs), with the surface multivalency of the viral material displaying specific antigens and enhancing the effective antigen delivery process by encapsulating adjuvants (Figure 6A) [101]. With the use of flash nanocomplexation (FNC) technology, they may be manufactured in large quantities and produce high titers of SARS-CoV-2 neutralizing antibodies, which in turn stimulate a powerful immune response that is protective. In addition to the membrane-encapsulated form, Zhang et al. created an inhalable hybrid nanovaccine with a viral bionic structure called NVRBD-MLipo. This was achieved through the effective fusion of genetically modified NVs expressing an RBD (called NVRBD) and MPLA-containing, PS-biomimetic liposomes (called MLipo), which have long-term stability in PBS or serum (Figure 6B) [19]. The creation of cell-derived nanovesicles (NVs) using ultrasound or cell membrane squeezing has garnered significant interest as a potentially effective means of enhancing vaccine delivery methods [115].

## 4. Conclusions

A significant advancement in the field of vaccination was triggered by the COVID-19 pandemic caused by SARS-CoV-2. It greatly accelerated the development and application of vaccine nanotechnology. Within two months of the SARS-CoV-2 genome’s publication, clinical trials for nanoparticle vaccine candidates commenced, a testament to decades of work in building novel vaccine platforms. This rapid response, combined with the high efficacy of the approved COVID-19 nanoparticle vaccine, saved countless lives. A pivotal moment in the history of vaccine development has arrived as the world struggles with the problems caused by infectious diseases, mainly due to the COVID-19 pandemic. However, nanovaccines face significant challenges in clinical trials, such as ensuring their safety amidst concerns over their potential toxicity and immune responses, determining the optimal dosing and delivery methods, scaling up production while maintaining quality, meeting rigorous regulatory standards, and addressing cost-effectiveness. Future research should focus on enhancing their safety profiles through advanced nanoparticle design, developing targeted delivery systems for improved antigen presentation, optimizing their immunogenicity with adjuvants, improving their long-term stability and storage, and conducting diverse population studies to validate their efficacy and safety. These efforts promise to advance nanovaccine development, potentially enhancing vaccines’ effectiveness and accessibility globally.

A variety of nanomaterials promise to redefine the benchmarks for vaccine stability, efficacy, and targeted delivery (Table 2). This is a revolutionary advancement in vaccine technology as researchers are utilizing the unique qualities of these materials to create vaccines that can precisely navigate through the intricate biological environments of the human body, ensuring that the therapeutic agent is delivered exactly where it is needed. In addition, the emergence of computational modeling and artificial intelligence in vaccine design has set the stage for the era of precision medicine; with these tools, it is possible to craft vaccine components and fine-tune antigen presentation and immunomodulation [116,117].

Functionalization research and applications based on nanovaccines provide numerous ways to enhance vaccines’ efficacy, expedite therapeutic medication delivery, and shield individuals against SARS-CoV-2 and its variations. This degree of accuracy and control maximizes the vaccine’s effectiveness against the virus and its numerous variations, providing the best possible protection with the lowest likelihood of side effects. With the potential to drastically reduce the time and expenses involved in vaccine development, this will have far-reaching effects and speed up the response to new health risks. In addition, by understanding and utilizing the unique immune profiles of each individual, vaccines can be created to trigger the best possible immune response that is protective, ensuring optimal health outcomes. For example, in cancer immunotherapy, these vaccinations can be designed to target certain tumor antigens, strengthening the body’s immune response against cancer cells [118,119]. Similarly, the rapid design and deployment of nanovaccines against emerging infectious diseases is an important tool in the global health arsenal, providing the flexibility needed to combat rapidly spreading pathogens. The development of mucosal vaccines has opened up new avenues of protection against respiratory infections, sexually transmitted diseases, and gastrointestinal pathogens, providing enhanced protection at the primary site of pathogen entry into the body. The integration of nanotechnology innovations with mucosal delivery routes, the expansion of clinical applications, and the promotion of strategic collaborations are critical to unlocking the full potential of these advanced vaccines. This is a promising strategy for developing nations or situations where non-invasive routes of administration are necessary [120,121]. In conclusion, the research on the functionalization of nanovaccines is promising and revolutionary.

We gathered the pertinent literature and data covering the most recent advancements in research up until June 2024, using search engines and databases like PubMed, Web of Science, etc. However, because COVID-19 research is moving so quickly, more information and conclusions might become available after this study is published. Despite our best efforts, we may not have covered all of the main types of nanoparticles due to time and resource restrictions, especially with regard to developing or less-studied types. With this material, we intend to give readers a clearer understanding of the study’s limits and a more thorough background on the research, as well as aid in understanding the trustworthiness and application of the findings.

## Figures and Tables

**Table 1:** Nanoparticle-based vaccines that have been approved or successfully tested in clinical trials.

Vaccine	Nanoparticle Type	Disease Target	Stage of Development	Outcome/Notes
Pfizer-BioNTech COVID-19 Vaccine (Comirnaty)	Lipid Nanoparticles (LNPs)	COVID-19	Approved	Highly effective in preventing COVID-19, widely used globally.
Moderna COVID-19 Vaccine (mRNA-1273)	Lipid Nanoparticles (LNPs)	COVID-19	Approved	Demonstrated high efficacy in preventing COVID-19 infection.
Novavax COVID-19 Vaccine (NVX-CoV2373)	Protein-Based Nanoparticles	COVID-19	Approved (in some countries)	Protein subunit nanoparticles with adjuvant, effective in preventing COVID-19.
Flublok Quadrivalent Influenza Vaccine	Recombinant Hemagglutinin (rHA) Nanoparticles	Influenza	Approved	First influenza vaccine using recombinant technology, effective against influenza strains.
Gardasil 9	Virus-Like Particles (VLPs)	Human Papillomavirus (HPV)	Approved	Prevents HPV infection and associated cancers, including cervical cancer.
Cervarix	Virus-Like Particles (VLPs)	Human Papillomavirus (HPV)	Approved	Provides protection against HPV types 16 and 18, significant for cervical cancer prevention.

**Table 2:** An overview of the types of nanoparticles based on factors including the targeted approach, specificity, stability, and efficacy.

Type of Nanoparticle	Efficacy	Stability	Specificity	Targeted Approach
Lipid Nanoparticles (LNPs)	High efficacy, especially with mRNA vaccines in generating nAb	High stability, especially with PEGylation and ionizable lipids	High specificity with potential for targeting of specific cells or organs	Surface modification with ligands, antibodies, or aptamers; encapsulating targeting molecules
Inorganic Nanoparticles	Effective in eliciting strong immune responses through immunomodulatory properties	Stable with controlled release capabilities	High specificity due to the ability to functionalize surfaces	Functionalization with peptides, antibodies, or small molecules; surface coating with polymers
Protein-Based Nanoparticles	Effective in increasing the quality and quantity of immune protection	Generally stable with the potential for controlled production processes	High specificity by mimicking viral proteins and targeted delivery	Genetic engineering to include targeting moieties; conjugation with antibodies or specific receptor ligands
Biomimetic Nanoparticles	High efficacy in mimicking pathogen properties while maintaining nanoparticle stability	High stability and potential for long-term antigen and adjuvant protection	Very high specificity due to mimicking pathogen properties and targeted delivery	Coating with cell membranes from target cells; use of natural targeting ligands

## Data Availability

No new data were created or analyzed in this study.

## Abbreviations

SARS-CoV-2	Severe Acute Respiratory Syndrome Coronavirus 2
COVID-19	Coronavirus Disease 2019
NPs	Nanoparticles
LNPs	Lipid Nanoparticles
nAb	Neutralizing Antibody
NVs	Nanovaccines
SLNs	Solid Lipid Nanoparticles
NLCs	Nanostructured Lipid Carriers
PEG	Polyethylene Glycol
SPANs	Self-Assembling Protein Nanoparticles
VLPs	Virus-Like Particles
HBV	Hepatitis B Virus
HIV	Human Immunodeficiency Virus
mRNA	Messenger Ribonucleic Acid
RBD	Receptor-Binding Domain
APCs	Antigen-Presenting Cells
CpG	Cytosine-Phosphate-Guanine
MSNs	Mesoporous Silica Nanoparticles
CDNs	Cyclic Dinucleotides
STING	Stimulator of Interferon Genes
DCs	Dendritic Cells
ROS	Reactive Oxygen Species
NLRP3	NLR Family Pyrin Domain Containing 3
IgG	Immunoglobulin G
RNA	Ribonucleic Acid
DNA	Deoxyribonucleic Acid
